# Amyloid Formation by Globular Proteins: The Need to Narrow the Gap Between *in Vitro* and *in Vivo* Mechanisms

**DOI:** 10.3389/fmolb.2022.830006

**Published:** 2022-02-14

**Authors:** Giulia Faravelli, Valentina Mondani, P. Patrizia Mangione, Sara Raimondi, Loredana Marchese, Francesca Lavatelli, Monica Stoppini, Alessandra Corazza, Diana Canetti, Guglielmo Verona, Laura Obici, Graham W. Taylor, Julian D. Gillmore, Sofia Giorgetti, Vittorio Bellotti

**Affiliations:** ^1^ Unit of Biochemistry, Department of Molecular Medicine, University of Pavia, Pavia, Italy; ^2^ Wolfson Drug Discovery Unit, Division of Medicine, Centre for Amyloidosis and Acute Phase Proteins, University College London, London, United Kingdom; ^3^ Department of Medicine (DAME), University of Udine, Udine, Italy; ^4^ Istituto Nazionale Biostrutture e Biosistemi, Rome, Italy; ^5^ Amyloidosis Research and Treatment Centre, Fondazione IRCCS Policlinico San Matteo, Pavia, Italy; ^6^ National Amyloidosis Centre, University College London and Royal Free Hospital, London, United Kingdom; ^7^ Scientific Direction, Fondazione IRCCS Policlinico San Matteo, Pavia, Italy

**Keywords:** amyloidosis, transthyretin, β2-microglobulin, metamorphosis, amyloid

## Abstract

The globular to fibrillar transition of proteins represents a key pathogenic event in the development of amyloid diseases. Although systemic amyloidoses share the common characteristic of amyloid deposition in the extracellular matrix, they are clinically heterogeneous as the affected organs may vary. The observation that precursors of amyloid fibrils derived from circulating globular plasma proteins led to huge efforts in trying to elucidate the structural events determining the protein metamorphosis from their globular to fibrillar state. Whereas the process of metamorphosis has inspired poets and writers from Ovid to Kafka, protein metamorphism is a more recent concept. It is an ideal metaphor in biochemistry for studying the protein folding paradigm and investigating determinants of folding dynamics. Although we have learned how to transform both normal and pathogenic globular proteins into fibrillar polymers *in vitro*, the events occurring *in vivo*, are far more complex and yet to be explained. A major gap still exists between *in vivo* and *in vitro* models of fibrillogenesis as the biological complexity of the disease in living organisms cannot be reproduced at the same extent in the test tube. Reviewing the major scientific attempts to monitor the amyloidogenic metamorphosis of globular proteins in systems of increasing complexity, from cell culture to human tissues, may help to bridge the gap between the experimental models and the actual pathological events in patients.

## Introduction

**FIGURE 1:**
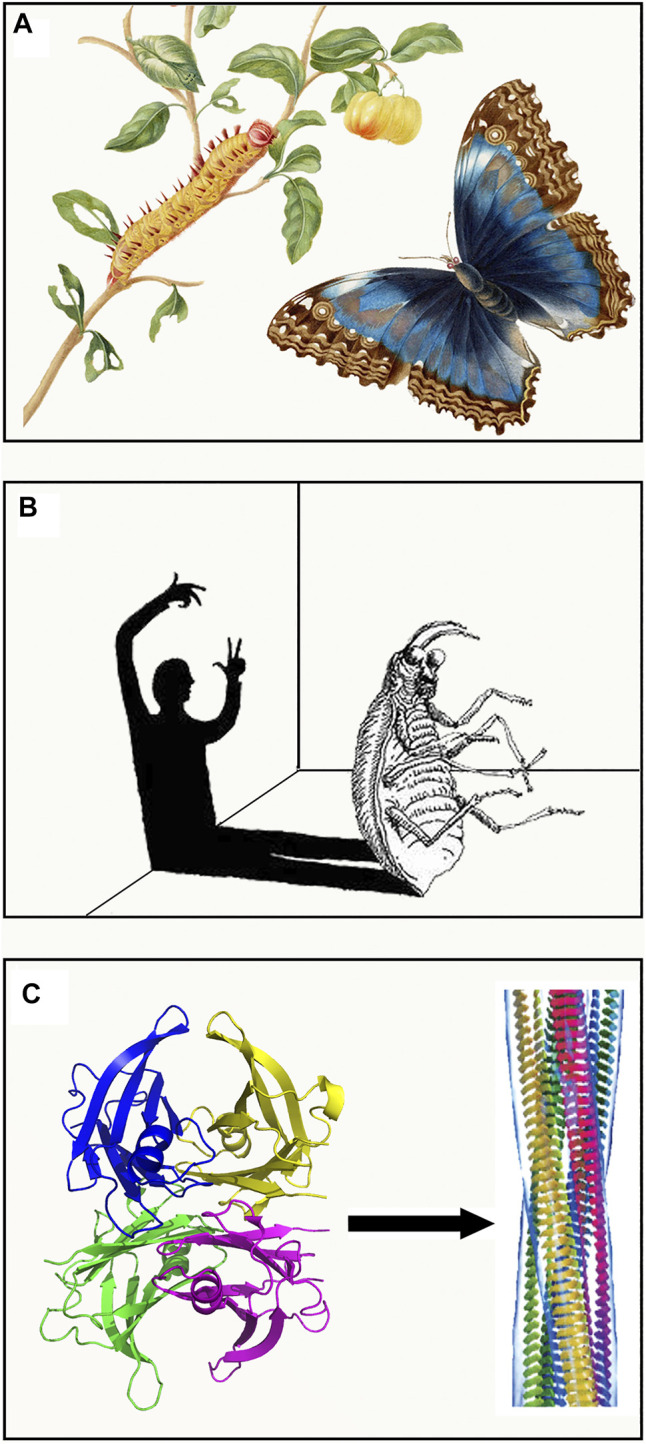
Methamorphosis in nature and literature. **(A)** The caterpillar and butterfly captured by the scientific illustrator and naturalist Maria Sibylla Merian (1647–1717). **(B)** Representation of the nightmare in which Gregory Samsa is being transformed from human to a beetle. **(C)** The conversion of native tetrameric TTR into amyloid fibrillar structures marks the protein transition from a physiological to a pathologic role. Both artworks in **(A,B)** are in the public domain. Structure of amyloid fibrils from (Chiti and Dobson, 2017) are reprinted with permission from Annual Reviews, Copyright (2017).

**FIGURE 2:**
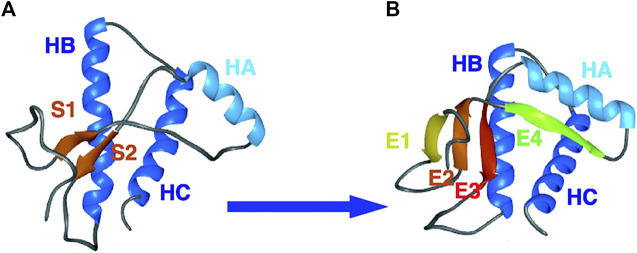
Simulated conversion of Syrian hamster D147N PrP^C^ to PrP^Sc^ at low pH levels. The wild-type NMR structure is shown **(A)** with the helices and strands labeled. A representative PrP^Sc^-like structure is shown **(B)**. Adapted from (DeMarco and Daggett, 2004), Copyright (2004) National Academy of Sciences, U.S.A.

Metamorphosis is a process of transformation of an entity into a different form, acquiring a different identity. Although the two entities are different, their basic substance is identical: the caterpillar and butterfly have the same genome, and the humanity of “Gregor Samsa” is unchanged whether caged in human form or in the semblance of a beetle. In the same way, proteins with the same primary sequence (substance) can assume two completely different shapes. Metamorphosis is an intrinsic property of living creatures. It represents a fascinating phenomenon that has extraordinary evolutionary implications which increase the adaptation of living organisms to different environments and reduce competition for resources (Laudet, 2011). Entire generations of biologists have investigated this phenomenon which has, at the same time, stimulated imagination, dreams and artistic creativity. Metamorphosis has always inspired human imagination raising ambivalent sentiments from fear to amazement. Humankind have viewed the metamorphosis as an awe-inspiring biologic phenomenon (from the caterpillar to butterfly) (Figure 1A) or as a terrible nightmare (in the dream of being transformed into a horrible insect) (Figure 1B). More recently, the terms metamorphosis or metamorphism have been associated to the properties of particular proteins that may acquire multiple functions, such as moonlighting proteins (Dishman and Volkman, 2018) (http://www.moonlightingproteins.org), or exhibit different physiological and pathologic roles (Figure 1C). The prion protein represents an example of protein metamorphism in which the same protein sequence can adopt both a native globular fold (PrP^C^) and a self-propagating infectious one (PrP^Sc^) which is responsible for transmissible spongiform encephalopathies. The first structure of PrP^C^ was obtained by NMR (Riek et al., 1996) and shows a predominant alpha helical content (Figure 2). A model based on molecular dynamics simulations suggests that a high proportion of beta structures may characterize the conformation of the infectious prion. The mechanism by which the contact between the two isoforms triggers the conformational shift PrP^C^ to PrP^SC^ is not fully clarified yet, however specific contact sites suitable for triggering such a transition have been extensively studied (Brunori, 2021). A recent cryo-EM structure of a patient-derived amyloid fibril of PrP has provided highly resolved constraints that may shed light on a future structure-based understanding of prion biology (Kraus et al., 2021).

## Amyloidosis Is an Aging Associated Disease

Whilst systemic amyloidosis has traditionally been associated with the presence of destabilized protein variants, such as lysozyme or transthyretin (TTR), or markedly increased plasma protein concentrations (SAA) (Merlini and Bellotti, 2003), epidemiological studies have shown that there is also an association of amyloid with aging (Blumenthal, 2004; Trigo et al., 2019; Tasaki et al., 2021) (Table 1). This is particularly true for amyloid diseases caused by normal circulating levels of wild type proteins such as TTR (Nativi-Nicolau et al., 2021). In the last decade, the comparative analysis of hereditary versus non-hereditary amyloidosis has represented an extraordinary source of information for the intrinsic amyloidogenic properties of these proteins. The age of onset of the genetic forms is, most often, much earlier compared with those caused by wild type proteins thus revealing that single residue changes, even if hidden within the protein structure, can strongly enhance the amyloidogenic propensity of the protein (Table 1). The metamorphic property of some globular proteins is particularly intriguing in cases where the whole protein can be converted into the amyloid fibrils, presenting two totally different shapes despite a completely identical sequence. Among globular proteins, investigated for their metamorphic and pathogenic behavior, we have extensively studied human β2-microglobulin (β2-m) and TTR. We believe that the wealth of information acquired on structure and folding dynamics of both proteins is extremely informative on the generic molecular basis of protein amyloidogenesis. Both proteins are assembled in the early phase of their life into their quaternary structure: β2-m as a light chain of the major antigen of histocompatibility type I (MHCI) and TTR as a homotetramer which forms a complex with the retinol binding protein (RBP). Both proteins can cause amyloid in either their wild type form or in their genetic variants associated with the familial forms of the disease.

**TABLE 1:** Proteins that can cause amyloid deposits in humans either in their wild type form or in presence of mutations.

References	Fibril protein	Precursor protein	Age at onset	Acquired (A) or hereditary (H)	Target organs (clinically manifested)
(Merlini et al., 2018; Muchtar et al., 2019; Benson et al., 2020; Muchtar et al., 2021)	AL	Immunoglobulin light chain, monoclonal	Median 63 years	A	All organs, except CNS
Benson et al. (2015)		Immunoglobulin κ light chain (C_L_ domain)	n.a. (median age of end-stage renal disease 60 years)	H	Kidney
(Grogan et al., 2016; Muchtar et al., 2021; Nativi-Nicolau et al., 2021)	ATTR	TTR, wild type	Median age 70 years	A	Heart mainly in males, lung, ligaments, tenosynovium
Barroso et al. (2015)		TTR, variants	Mutation-dependent (median 39 years)	H	PNS, ANS, heart, eye, leptomeninges
(Gejyo et al., 1985; Stoppini and Bellotti, 2015)	β2-m	β2-m, wild type	n.a. (depending on dialysis onset)	A	Musculoskeletal system
(Valleix et al., 2012; Mizuno et al., 2021)		β2-m, variant(s)	Median age 50 years	H	GI, ANS, tongue, salivary glands
Koedam et al., (2010)	Aβ	Aβ protein precursor, wild type	>65 years	A	CNS
Latimer et al. (2021)		Aβ protein precursor, variant	<65 years (30 years–40 years)	H	CNS
Puoti et al. (2012)	APrP	Prion protein, wild type	55–75 years	A	CJD, fatal insomnia
Schmitz et al. (2017)		Prion protein variants	mutation-dependent (30–70 years)	H	CJD, GSS syndrome, fatal insomnia
(Mucchiano et al., 2001; Tasaki et al., 2021)	AApoAI	ApoAI, wild type^a^	Aging-associated	A	Aortic intima, carotid artery, meniscus with knee osteoarthritis, lumbar ligamentum flavum
Muchtar et al. (2021)		ApoAI, variants	Mutation-dependent (20–70 years)	H	Heart, liver, kidney, PNS, testis, larynx (C terminal variants), skin (C terminal variants)

C_L_, light chain’s constant domain; ApoAI, Apolipoprotein AI; GI, Gastrointestinal Tract; CNS, Central Nervous System; PNS, Peripheral Nervous System; ANS, Autonomic Nervous System; CJD, Creutzfeldt Jakob Disease; GSS, Gerstmann-Sträussler-Scheinker syndrome; n.a. not available.

a ApoAI WT is not listed as an amyloidogenic protein in the Amyloid nomenclature 2020 (Benson et al., 2020).

## Genetic Versus Acquired β2-m Related Amyloidosis

**FIGURE 3:**
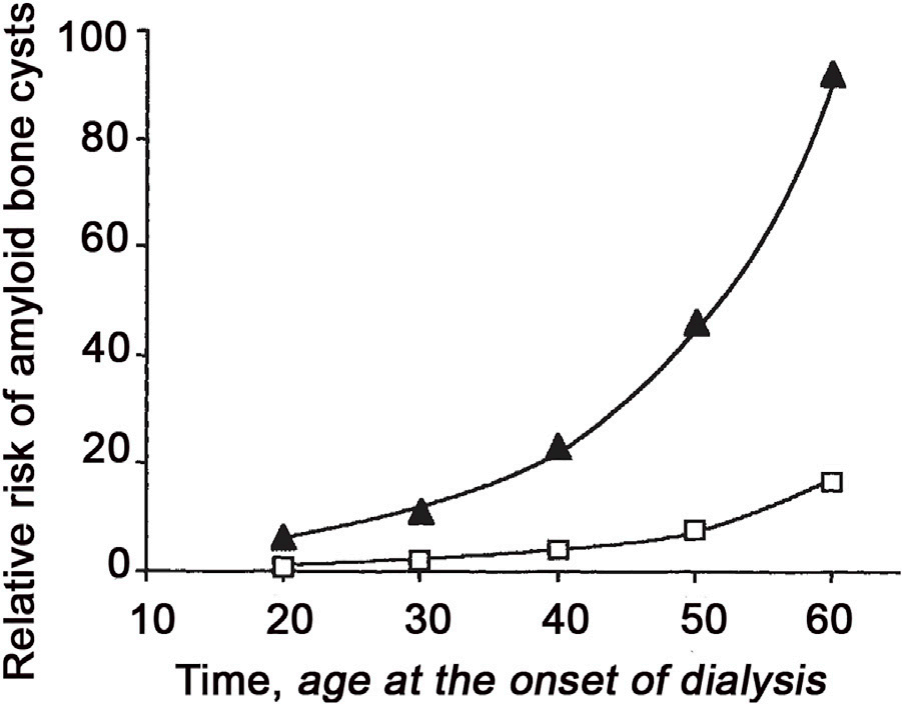
Relative risk of bone lesions by amyloid deposition as a function of age at the initiation of dialysis either on AN69 or a cellulosic membrane. The risk of developing the lesion with AN69 for patients starting at age 20 is assumed to be one. Data are based on the best fitted model provided by Van Ypersele de Strihou work (de Strihou et al., 1991). Reprinted from (de Strihou et al., 1991) with permission from Elsevier, Copyright (1991).

In 1985 the Japanese group of Prof Gejyo identified β2-m as the protein responsible for a peculiar form of amyloidosis (Gejyo et al., 1985) observed in the musculoskeletal system of patients under long term haemodialysis (Assenat et al., 1980). In patients under chronic haemodialysis, β2-m circulates at a concentration 5–20 fold higher that in normal subjects due to the limited efficacy of dialytic membranes to efficiently remove β2-m which, upon dissociation of MHCI, is continuously released from the surface of cells into circulation. The persistently high concentration of plasma β2-m is an essential requirement for the formation of amyloid *in vivo*, but other factors can modulate individual susceptibility to form amyloid deposits. Improved biocompatibility of dialytic membranes can reduce the inflammatory response and delay the onset of amyloid deposition. The physiological state of patients, and particularly their age, can affect their individual susceptibility to amyloid complication in hemodialysis. Van Ipersele de Strihou and his group has clearly shown that the risk of developing amyloid complications exponentially increases with the age of the patient at the onset of the dialysis treatment (Figure 3) (de Strihou et al., 1991). This finding is important because Dialysis Related Amyloidosis (DRA) is probably the only amyloid disease in which we can exactly establish the time (the first day of haemodialysis) in which a pro-amyloidogenic phase is triggered by simply increasing the concentration of plasma β2-m. In the last few decades, the intermediate steps along the pathway of β2-m amyloid formation *in vitro* have been identified (Michaels et al., 2018). It is plausible that primary nucleation, fragmentation, surface-catalyzed secondary nucleation, elongation and dissociation of fibrils can determine the rate of amyloid growth *in vivo*, but it is not known how the particular biological environment, within a living organism, could affect these intermediate steps. Even more challenging is to elucidate how aging can influence the rate of the intermediate steps of fibrillogenesis *in vivo*. There are several working hypotheses on the role of aging in amyloidogenesis: malfunction of proteostasis (Trigo et al., 2019), modification of the extracellular matrix (Morawski et al., 2014) and, impairment in the bio-energetic efficiency (Swerdlow, 2018) are among the most accredited theories. The molecular transition of β2-m from its native to fibrillar conformation suggests that partial unfolding is required to prime aggregation. Early studies were carried out *in vitro* at a low pH revealing that fibrillogenesis of β2-m requires the unfolding of a large portion of the protein. Conditions used in the early studies to generate *in vitro* amyloid fibrils, both in the case of β2-m and, in general, for almost all the globular proteins including lysozyme, the archetypal protein of these studies (Booth et al., 1997), were not compatible with the physicochemical characteristics of the biologic environment even those of an aged organism. However, there was evidence that natural fibrils contained not only the full length β2-m, but also a significant proportion of a truncated species lacking 6 residues at N-terminal end (Bellotti et al., 1998). Further studies confirmed that the N-terminal truncation reduced the β2-m folding stability and generated fibrils even under a more physiological environment (Esposito et al., 2000). The metamorphism of the protein was somehow triggered by a selective proteolytic cleavage. This suggests that proteolysis-mediated remodeling may either be a physiological mechanism for priming β2-m degradation, or a purely a pathologic processing event enhanced in hemodialysis and, in particular, in elderly patients. The question remains unanswered due to the complexity of the system. Indeed, to prevent the adverse effects of dialysis related amyloidosis, more efforts have been devoted to ameliorate hemodialytic procedures rather than to discover drugs targeting the key steps of fibril formation. Our experience suggests that curiosity-driven programs in the biomedical field, more and more often, compete with translational projects that aim to solve medical needs diminishing the resources and efforts to elucidate the biological mechanisms underlying the disease and discover new therapeutic targets. Nevertheless, seminal observations have enabled advances in the field.

The observation that minimal truncation of β2-m is sufficient to prime β2-m fibrillogenesis in bio-compatible conditions was further strengthened by the demonstration that, in a physiological environment, truncated β2-m can play a prion like effect on full length β2 (Karamanos et al., 2014). The mechanism requires the formation of fibrillar seeds by the truncated species thus generating a suitable fibrillar template which triggers a chain polymerization mix of non-covalently linked oligomers of full length and truncated species, both acquiring an “amyloid conformation” (Natalello et al., 2016). Discovery of the first genetic variant of β2-microglobulin (Valleix et al., 2012) confirmed that destabilization of the tertiary structure is a generic prerequisite for amyloid transformation and that fibrillar seeds have a potent, potential, role in the propagation of amyloid formation. We say “potential role” because, although D76N variant was clearly shown to be a potent driver for the conversion of wild type β2-m into an amyloid conformation (Mangione et al., 2013), we were unable to demonstrate the co-deposition of the wild type within the natural D76N fibrils extracted from the spleen of one patient. A second genetically transmitted amyloidogenic variant of β2-m (V27M) has been recently described (Mizuno et al., 2021). In this case, the *in vitro* studies suggest a striking similarity with the biophysical properties of the D76N variant, both showing a similar level of destabilization in comparison to the wild type, and both forming amyloid fibrils in physiological buffer and with similar morphology when analyzed by electron microscopy. However, the natural fibrils extracted from the hip joint synovium contained a combination of 25% wild type and 75% V27M variant. This suggests that the “prion like” effect, in which the variant acts as primer to convert the wild type protein into fibrils, might be different, depending on the specific isoform and type of tissue that may offer more or less protection to co-propagate heterogeneous isoforms. Characterization of natural fibrils derived from amyloidogenic β2-m variants with similar biophysical properties, isolated in subjects with different ethnicity and, affected by two different types of syndromes, dramatically reveal the complexity of the phenomenon in which multiple factors play a pathogenic role.

## Animal Models of β2-m

Several experimental models have been developed to investigate the molecular mechanisms of amyloidogenesis by human proteins *in vivo*, ranging from simple organisms such as *C. elegans*, to mammalians, such as mice and, even primates in rare cases. Our capacity to reproduce the clinical effects of β2-m in animal models, remains still a challenging task.

Particularly informative is the failure to create a mouse model of β2-m amyloidosis which was reported by Higuchi’s group (Zhang et al., 2010) in 2010. Despite an overexpression of human β2-m in a murine β2-m knockout background, no β2-m related amyloid was formed in those mice even at their later stage. Finally, the amyloid observed was caused by Apolipoprotein A2 (ApoA2) which spontaneously forms amyloid in the specific strains of mice used in that laboratory. Even the attempt to induce amyloid deposition by injecting seeds of natural human fibrils failed. Failure to induce β2-m amyloidosis in transgenic mice expressing the D76N β2-m variant (Halabelian et al., 2014) was even more surprising and, very informative at the same time, as this protein is highly amyloidogenic both *in vivo* and *in vitro* (Valleix et al., 2012). The use of strains knocked out for murine β2-m excludes a protective role of the endogenous β2-m that otherwise is known to interfere with the aggregation of the human counterpart (Eichner and Radford, 2011; Karamanos et al., 2014; Achour et al., 2020). The specific protection of rodents against β2-m related amyloidosis is due to unidentified factors. Although mice can develop some specific forms of murine amyloidosis (such as AAPOA2), either spontaneously or upon stimulation, they are extremely resistant to other types of systemic amyloidosis, and in particular, to the type caused by immunoglobulin light chains. The only mouse models in which amyloid deposition is easily and consistently achieved, under experimental conditions, are those related to HDL related lipoproteins. Persistently high concentration of serum Amyloid A (SAA), in the presence (McAdam and Sipe, 1976) or even in the absence of chronic inflammation (Simons et al., 2013), cause AA amyloidosis. On the other hand, ApoA2 causes a senescence-accelerated and spontaneous form of amyloidosis in which the rate of deposition depends on specific polymorphisms of the protein (Higuchi et al., 1991). Why mice are susceptible to some types of systemic amyloidosis and totally resistant to others is not understood, and highlights the complexity of the mechanism of systemic deposition of amyloid in a living organism and, in particular, in vertebrates. Regarding systems of amyloidosis in invertebrates, *C. elegans* is an extremely interesting and versatile model for the study of natural aging in a living organism (Mack et al., 2018). Pathogenic effects of human soluble and oligomeric species of β2-m have extensively reported for wild type species as well as for the clinically pathogenic variants. Although worm models cannot recapitulate the clinical and pathologic complexity of the human disease, they can certainly highlight and single out specific steps within the process. *C. elegans* was specifically chosen to represent the pathophysiology of β2-m related amyloidosis because, as with all the invertebrates, it lacks the MHCI complex and therefore β2-m is expressed and secreted as free monomer. In the first models of *C. elegans* expressing human β2-m, effects of different isoforms of the protein on survival and performance of different transgenic strains were described (Diomede et al., 2012). At a time in which the pathogenic variants of β2-m were not known yet, worms expressing the natural truncated form of wild type β2-m (ΔN6β2-m), were generated. It was shown that ΔN6β2-m was not only highly amyloidogenic *in vitro* (Esposito et al., 2000), but it had also a strong tendency to form oligomers in the worms affecting both their survival and movement performance.

It was not possible to demonstrate the formation of genuine amyloid fibrils, but the evidence of a pathologic phenotype associated to β2-m aggregation and toxicity, made the *C. elegans* model the only available *in vivo* system to study the putative pathogenic property of β2-m. The discovery of the first natural amyloidogenic variant of β2-m in members of a French family (Valleix et al., 2012) and, the demonstration of its higher propensity to make fibrils *in vitro* compared to the wild type, inspired the generation of a *C. elegans* strain expressing the D76N β2-m variant. Interestingly, the protein appeared to be highly toxic for the worms which died before reaching their first larval stage. The only way for them to reach adulthood was achieved by using a temperature-inducible system to switch-on the D76N β2-m expression only at their larval phase. The reason of such a high toxicity, incompatible with the worm survival in the larval phase is unknown; it represents a paradox in the context of a disease which is known to be strictly associated to aging, rather than to a very early phase of development.

A nematode model, established in another laboratory has fully validated previous data and further demonstrated that the expression of the highly amyloidogenic D76N β2-m and ∆N6 β2-m variants can cause global aggregation of endogenous *C. elegans* proteins at an advanced stage (Good et al., 2021). Disrupted ER homeostasis, combined with increased endogenous protein aggregation, were identified as drivers of β2-m associated toxicity *in vivo*. All the *C. elegans* β2-m models so far established display a reduced lifespan, impaired motility and developmental delays. Studies on the nematode models have also highlighted their importance as drug search tools. Use of a generic inhibitor of aggregation of amyloidogenic proteins, doxycycline, in the *C. elegans* strain expressing D76N β2m showed that the drug was able to rescue the adverse phenotype most likely by interfering with the formation of oligomeric conformers (Faravelli et al., 2019).

## Genetic Versus Acquired TTR Amyloidosis

**FIGURE 4:**
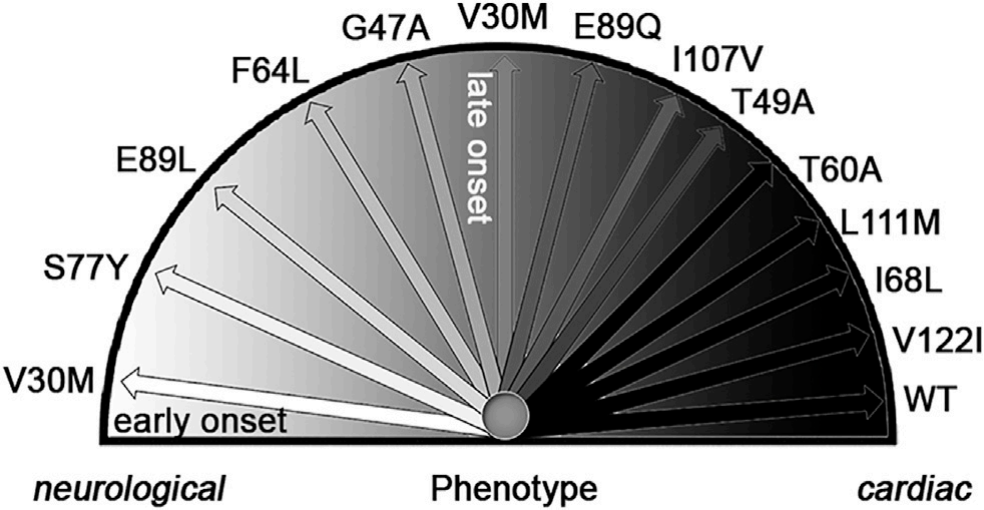
Genotype-phenotype correlation in ATTR amyloidosis. Adapted from Figure 2 in a previous publication by Rapezzi and collaborators (Rapezzi et al., 2013). Phenotypic expression of transthyretin-related amyloidosis varies widely from an almost exclusively neurological involvement (V30M mutation with early-onset disease) to a predominant or exclusively cardiac involvement (T60A, L111M, I68L, and V22I mutations and wild type). In between, several transthyretin-related amyloidosis mutations are associated with variable degrees of neurological and cardiac involvement, including V30M with late-onset disease. Adapted with permission by Oxford University Press, Copyright (2012).

**FIGURE 5:**
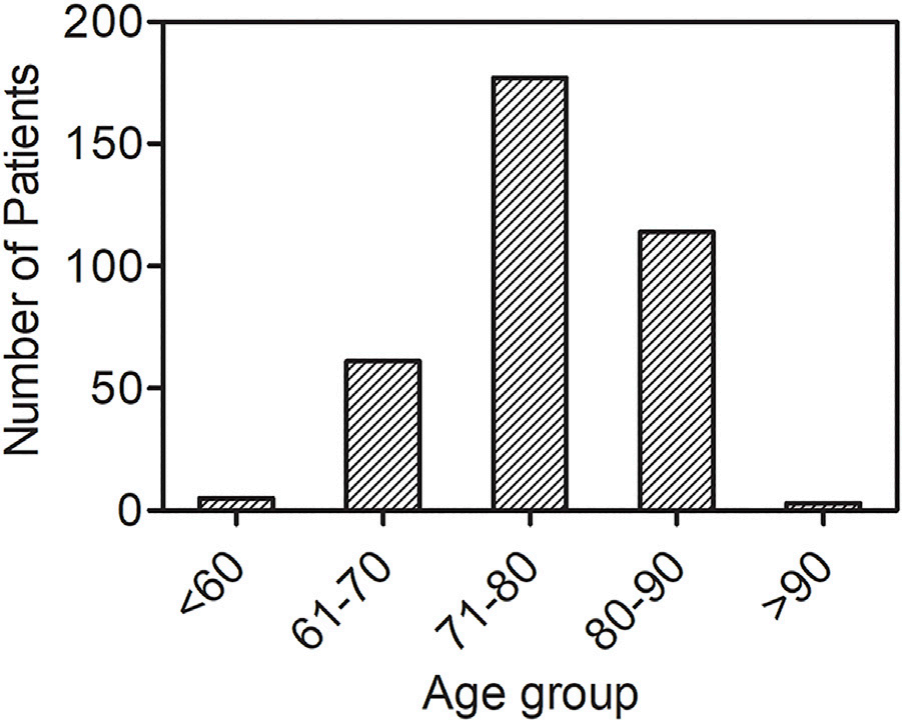
Number of patients diagnosed, *ante mortem,* for wild type ATTR. Data shown are based on a retrospective review of patients diagnosed with wild type ATTR at the Mayo Clinic through 2013 with the purpose of studying the natural history of the disease (Grogan et al., 2016).

**FIGURE 6:**
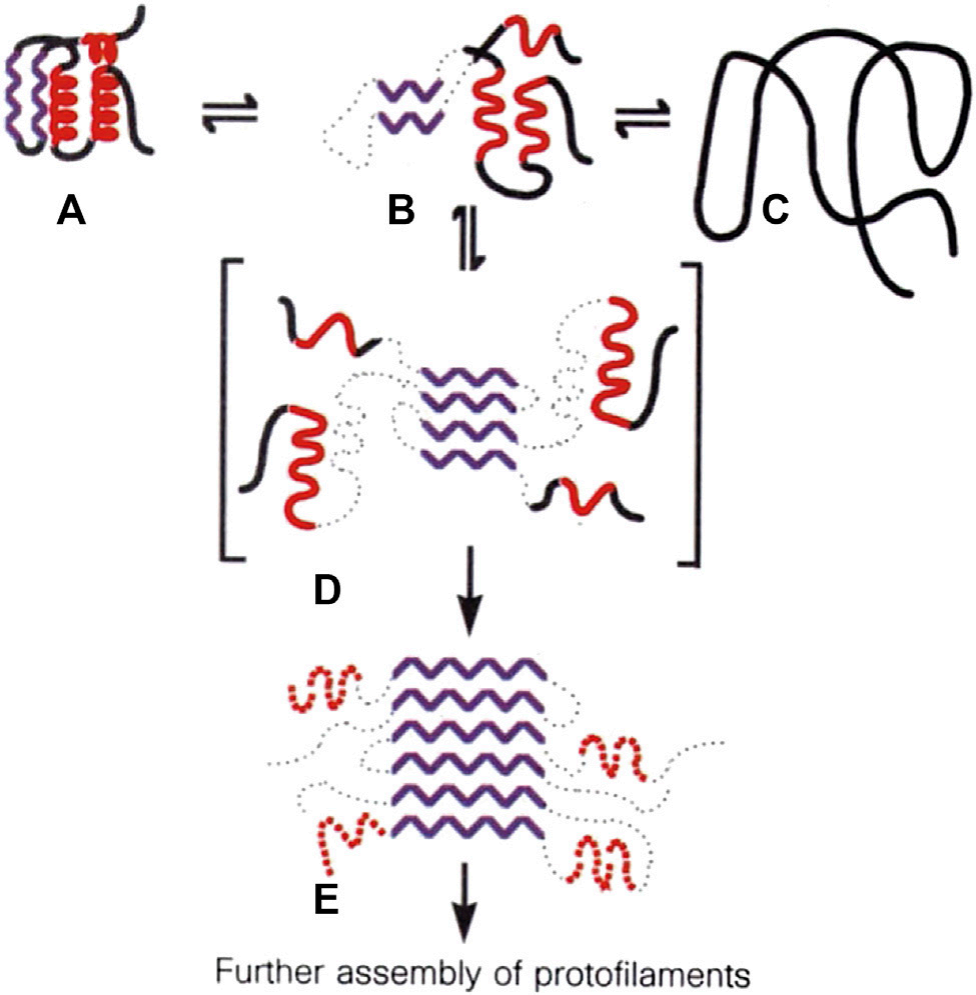
Proposed mechanism for lysozyme amyloid fibril formation. A molten globule form of the protein **(B)**, distinct from the native **(A)** and denatured **(C)** states, self-associates through the β-domain **(D)** to initiate fibril formation and form the core structure of the fibril **(E)**. The scheme was included in a previous manuscript published in Nature, 1997 (Booth et al., 1997).

**FIGURE 7:**
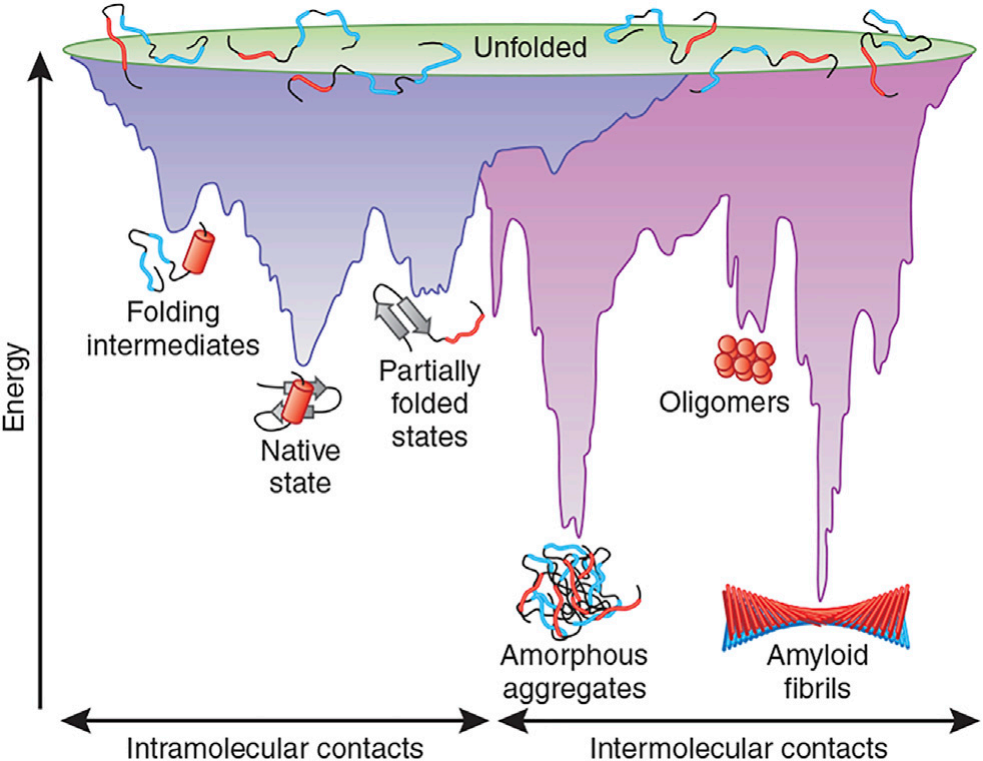
Energy landscape scheme of protein folding and aggregation. The scheme shows multiple conformations “funneling” to the native state *via* intramolecular contacts (purple surface) as well as conformations moving toward amorphous aggregates or amyloid fibrils *via* intermolecular contacts (pink area). Reprinted from (Hartl and Hayer-Hartl, 2009) with permission from Springer Nature Customer Service Centre GmbH, Springer Nature, Copyright (2009).

In 1978 the Portuguese group led by Pedro Costa demonstrated that Familial Amyloid Polyneuropathy (Andrade, 1952) was caused by the protein transthyretin (Costa et al., 1978) through a classical mechanism of autosomal dominant transmission (Saraiva, 2015). Histologically, TTR amyloid has been detected in all organs with the exception of brain parenchyma. After the discovery of the first amyloid associated mutation (V30M), more than one hundred amyloidogenic mutations have been identified (http://amyloidosismutations.com). Clinical pathologic features are well described with regard to the prominent involvement of one or multiple organs. Often the disease begins apparently with a single organ involvement and, in most cases, a systemic deposition of amyloid can further be observed. For example, following initial infiltration in the peripheral nerves with consequent polyneuropathy, amyloid deposition is frequently observed in the heart. There is a correlation between the type of single amino acid variant and the clinical phenotype. Some mutations cause a prominently or even an exclusively cardiac amyloidosis, whilst other mutations are primarily responsible for a polyneuropathic syndrome (Figure 4). Tissue specificity in systemic TTR amyloidosis sounds like a contradiction since it would be more appropriate to say that amyloid accumulates preferentially in some organs. In 1980 Sletten and Swedish colleagues (Sletten et al., 1980) showed that wild type transthyretin was also amyloidogenic and responsible for an already known non-familial form of cardiac amyloidosis mostly affecting the elderly population. Development of modern imaging techniques (Jurcuţ et al., 2020) has enabled accurate and early identification of cardiac TTR amyloid leading to a highly significant increase in the diagnosis of this disease. It is estimated that 25% of people over 75 are likely to have cardiac deposition of TTR amyloid with a consequent high probability of developing heart failure with preserved ejection fraction (HFpEF) (Grogan et al., 2016). The number of elderly patients affected by wild type TTR amyloidosis (Figure 5) suggests that the category of TTR amyloidosis is moving from a rare to a common disease. The urgent need to understand the pathogenic mechanism and establish effective therapies can now benefit from the extensive investigation carried out in the last decades on the rare amyloidogenic variants associated to the familial forms of the disease. In the early nineties, the Kelly’s group made the seminal observation that tetramer stability of TTR was impaired by the amyloidogenic mutations (Hammarström et al., 2002) and that the tetramer disassembly was a perquisite for the aggregation of TTR into an amyloid-like aggregates (Colon and Kelly, 1992). These observations were consistent with those made by the Wetzel’s group on amyloidogenic immunoglobulin light chain (Hurle et al., 1994), and the Pepys’s team on amyloidogenic lysozyme (Booth et al., 1997). The latter study highlighted the possibility of identifying a conformational intermediate along the folding pathway which was particularly susceptible to polymerization into a well-ordered cross-beta structure (Figure 6). After many decades of studies on the structural intermediates of the amyloidogenic pathway of TTR, we are still debating on the structure of misfolded monomers which lead to TTR fibrillogenesis. Nature offered an additional extraordinary tool for proving the causative role of folding destabilization in protein amyloidogenesis. Clinicians in Portugal observed that a small proportion of carriers of the amyloidogenic V30M mutation were protected from the disease and that the protection was provided by the concomitant T119M mutation in the TTR gene so that the resulting tetrameric protein was a mix of protomers presenting both the amyloidogenic V30M variant as well as the protective T119M. Demonstration that the presence of protomers with T119M has a stabilizing effect on the tetramer is a strong evidence in favor of the hypothesis that misfolding is at the basis of the disease. This widely accepted concept strongly encouraged the therapeutic strategy to stabilize TTR by small molecules. Colin Blake first showed that halogenated aromatic hydrocarbon can bind with high affinity to the thyroxine (T4) binding pocket of TTR thus designing the first study of the structurally-based design of TTR ligands (Rickenbacher et al., 1986). T4 and analogues became instrumental to the discovery of druggable stabilizers of the native tetrameric structure (Connelly et al., 2010) thus mimicking the enhanced stability observed in the presence of the T119M mutation. After almost 30 years, the approach of stabilizing the tetramer by a ligand has become a clinical reality. Tafamidis is now in the market for the treatment of TTR related amyloidoses and other compounds are in clinical trials such as acoramidis (Judge et al., 2019) or in the pipeline for being tested in clinical studies (Corazza et al., 2019). In the field of amyloidoses, it represents the most advanced experience of tackling disease by directly interfering with the misfolding mechanism. The discovery of drugs improving the stability of TTR highlights the importance of understanding more about the forces that can determine the TTR tetramer disassembly *in vivo*. Although it is well-known that destabilization is a pre-requisite for priming the amyloid pathway, little is known about the TTR misfolding energy landscape (Hartl and Hayer-Hartl, 2009) (Figure 7). Our efforts in looking for this source of energy were rewarded when we found out that biomechanical forces can provide sufficient energy to unfold the amyloidogenic proteins. Interestingly, these results represent a typical case of cross-fertilization between studies carried out in parallel on β2-m and TTR. The first observation on the capacity of biomechanical forces to misfold an amyloidogenic protein was reported for the characterization of the first pathogenic variant of β2-m. Later, this concept was applied to TTR amyloidogenesis, and, in this case, we discovered that local destabilization makes the protein more susceptible to cleavage by specific proteases. Proteolytic remodeling of protein exposed to biomechanical forces is a well-established process of great functional impact. The best example of mechano-enzymatic mechanism was described for the Von Willebrand factor (Zhang et al., 2009), where altering the protein structure *in vivo* can lead to both a positive and negative effect. Indeed, a specific genetic mutation either of the von Willebrand gene or of its specific protease, ADAMTS13 (Crawley et al., 2011), can respectively reduce or enhance the proteolytic activity. The system shifts from a sophisticated physiological control of hemostasis into a thrombotic or hemorrhagic syndrome. The mechano-enzymatic mechanism of TTR cleavage that our group has characterized in an *in vitro* and in an *in vivo* model of amyloidogenesis may be crucial in the pathogenesis of TTR related amyloidosis and, in particular, for the deposition of amyloid in organs where the biomechanical forces are particularly intense such as the heart and carpal tunnel. However, it is not known if it may have any physiologic role in the normal TTR catabolism.

## Animal Models of TTR

More than 10 years after the discovery that the V30M variant TTR was the causative protein of familial amyloid polyneuropathy, the first mouse model of TTR amyloidosis was published by the Araki’s group (Yi et al., 1991). Despite the presence of amyloid in the very old mice, the attempt to reproduce the clinical and pathologic features of amyloid polyneuropathy similar to the human counterpart was unsuccessful. This first model was followed by many other transgenic strains extensively reviewed by Buxbaum in 2009 (Buxbaum, 2009). Mice expressing other human TTR variants were explored and, in particular, the highly unstable and clinically very aggressive L55P, and the I84S in which the mutation prevents the formation of the complex TTR-RBP. It is worth noting that only one third of mice expressing the L55P variant in a null-TTR background presented amyloid deposition (Sousa et al., 2002). When mice expressing V30M were crossed with those lacking the main heat shock transcription factor (Hsf1), Santos and collaborators observed amyloid deposits in mice at younger age with TTR deposits in sciatic nerve and dorsal root ganglia thus showing a striking similarity with the human disease (Santos et al., 2010). The mechanism by which Hsf1 can protect from amyloid deposition is still uncertain because this transcription factor has multiple beneficial effect on proteostasis, either directly inhibiting the TTR aggregation through specific chaperones or protecting the target tissue from amyloid toxicity. This mouse model, however, represents the first successful example of the *in vivo* multifactorial modulation of TTR amyloidosis. The complexity of the process of *in vivo* amyloidogenesis is highlighted by other two mouse models more recently described. Li and colleagues (Li et al., 2018) have successfully produced humanized mouse strains at both the *Ttr* and *Rbp4* loci, allowing human TTR (hTTR) to associate with human retinol binding protein 4 (hRPP4). Remarkably, these animals develop age-related amyloid deposits mainly in the peripheral nerves and gastro-intestinal tract. This model narrows the gap between experimental and clinical neuropathology since the deposition of amyloid affects the perineurium of sciatic nerve that was only previously achieved in TTR transgenic mice which did not express Hsf1 (Santos et al., 2010). The role of the V30M pathogenic mutation on amyloid formation was not so remarkable. In fact, apparently the onset of amyloid deposition was more anticipated in the wild type (V30) than in the variant (M30) strain, however, it was more abundant in elderly mice carrying the TTR variant (M30). All those mouse models developed a very limited amount of amyloid in the heart in which non-fibrillar amorphous aggregates of TTR were mostly observed. The deposition in the heart has been very recently obtained in a new transgenic mouse strain expressing the human S52P TTR variant in which the formation of amyloid requires pre-inoculation with seeds of natural amyloid fibrils (Slamova et al., 2021). Here, the time frame of the onset of amyloid deposition and the amount of deposits are strictly dependent on the plasma concentration of TTR and, the amyloid mainly affects the heart and tongue. Interestingly, inhibition of the expression of α2-antiplasmin significantly anticipates the amyloid formation and aggravates the amyloid load, thus highlighting the possible pathogenic role of plasmin (Mangione et al., 2018).

In parallel to the development of disease models in mice, other interesting systems of TTR expression have been recently generated using *C. elegans* and *Drosophila melanogaster*. Nematode strains expressing wild type TTR, V30M TTR, D18G TTR and the non-aggregation-prone T119M TTR in the body-wall muscle have been generated (Madhivanan et al., 2018); among these, the ATTR V30M and D18G mutants displayed the accumulation of insoluble forms of TTR. Proteotoxicity, caused by each of the pathogenic TTR mutations, was monitored in the very short time frame of the worm’s life (3 weeks). In particular, the expression of TTR variants resulted in defects in neuronal morphology, nociceptive response and locomotion of the nematodes. Tsuda et al. (2018) have confirmed that *C. elegans* is an excellent tool to investigate toxicity of TTR conformers with particular regard to truncated species which are abundant in natural fibrils (Bergstrom et al., 2005; Ihse et al., 2013) and highly fibrillogenic *in vitro* (Mangione et al., 2014). The transgenic strains expressing the 49–127 and 81–127 TTR fragments showed a reduced motility and a significantly shortened lifespan compared with those expressing the full-length protein.

Transgenic flies have been used to study familial amyloidotic polyneuropathy. *Drosophila* expressing the amyloidogenic L55P TTR and the engineered variant TTR-A (V14N/V16E TTR) have been generated and compared with flies expressing wild type TTR. Both variants exhibited time-dependent aggregation of misfolded TTR, neurodegeneration, shortened lifespan, and compromised flying ability (Pokrzywa et al., 2007). In contrast, flies expressing wild type TTR did not show any effect on either longevity or behavior. Expression of the V30M TTR variant in flies led to similar outcomes associated to neurological impairment with evidence of Congo red positive deposits in the brain of the aged transgenic flies (Berg et al., 2009). Studies conducted on transgenic strains of *drosophila* led also to the identification of peptides as potential inhibitors of TTR aggregation as they were able to delay ATTR progression in the flies whilst decreasing TTR deposition (Saelices et al., 2018).

**FIGURE 8:**
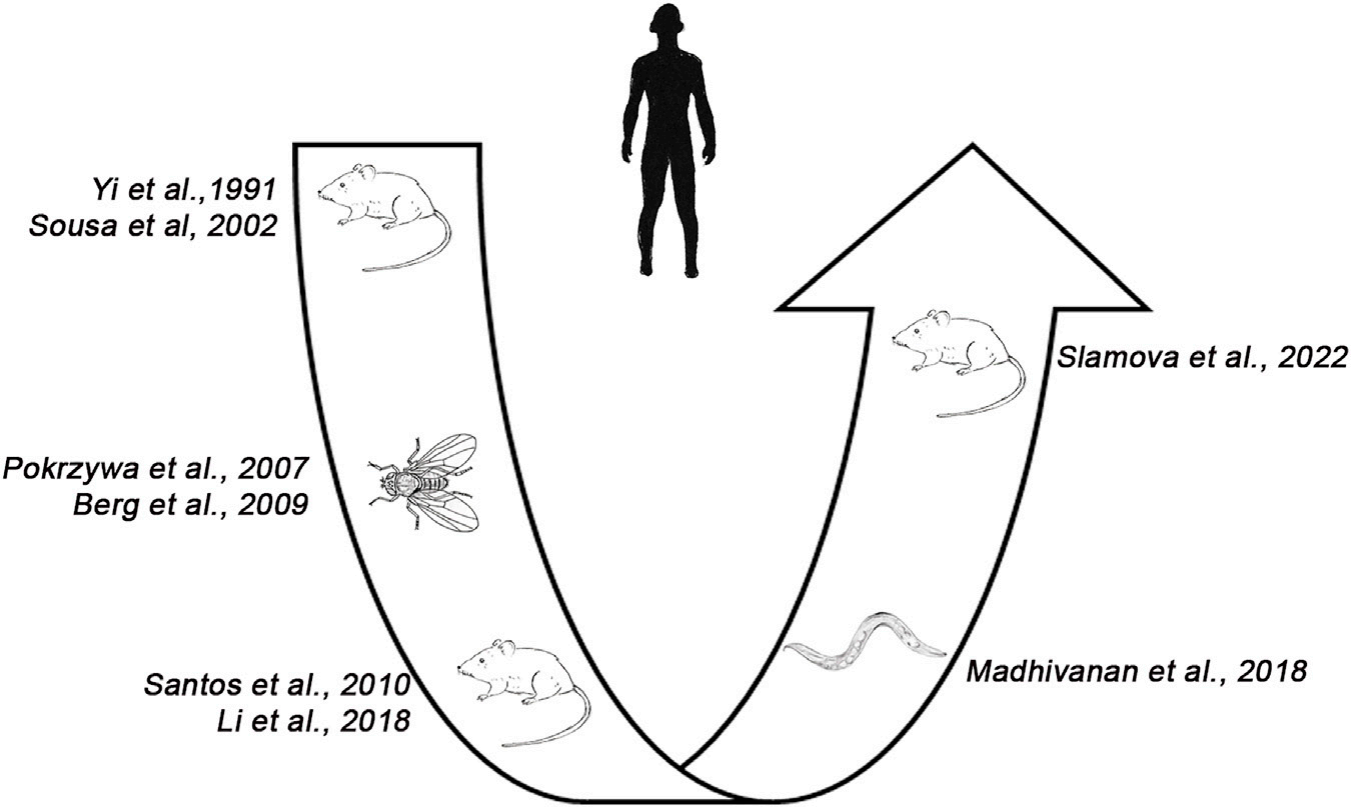
Development of animal models for ATTR in humans. From the first mouse model (Yi et al., 1991), several attempts were made to reproduce the disease in invertebrates (i.e., *C. elegans*, *Drosophila melanogaster*) before developing a novel transgenic mouse model reproducing amyloid in the heart and tongue (Slamova et al., 2021).

Looking at the development of animal models for TTR related amyloidosis over the years, (Figure 8) it appears that we moved from complicated living organisms (i.e., mice) to quite simpler invertebrate models, such as *C. elegans* and *Drosophila* and, finally back to mice.

Nevertheless, we must also mention the extraordinarily informative primate counterpart of human disease, that cannot be considered a model. It is actually a disease that affects other species very close to humans in which aging and individual susceptibility play similar roles (Nakamura et al., 2008; Chambers et al., 2010; Ueda et al., 2012).

## Conclusion

Despite extraordinary progress made in the last 3 decades on the theoretical and experimental elucidation of the molecular basis of metamorphogenesis of amyloidogenic proteins, we still do not understand which are the major molecular events that *in vivo* dictate the transformation of globular proteins into fibrils. The progress in the field has resulted in a challenge to Anfinsen’s paradigm that, under physiological conditions, a protein’s final structure is only determined by its primary sequence.

**FIGURE 9:**
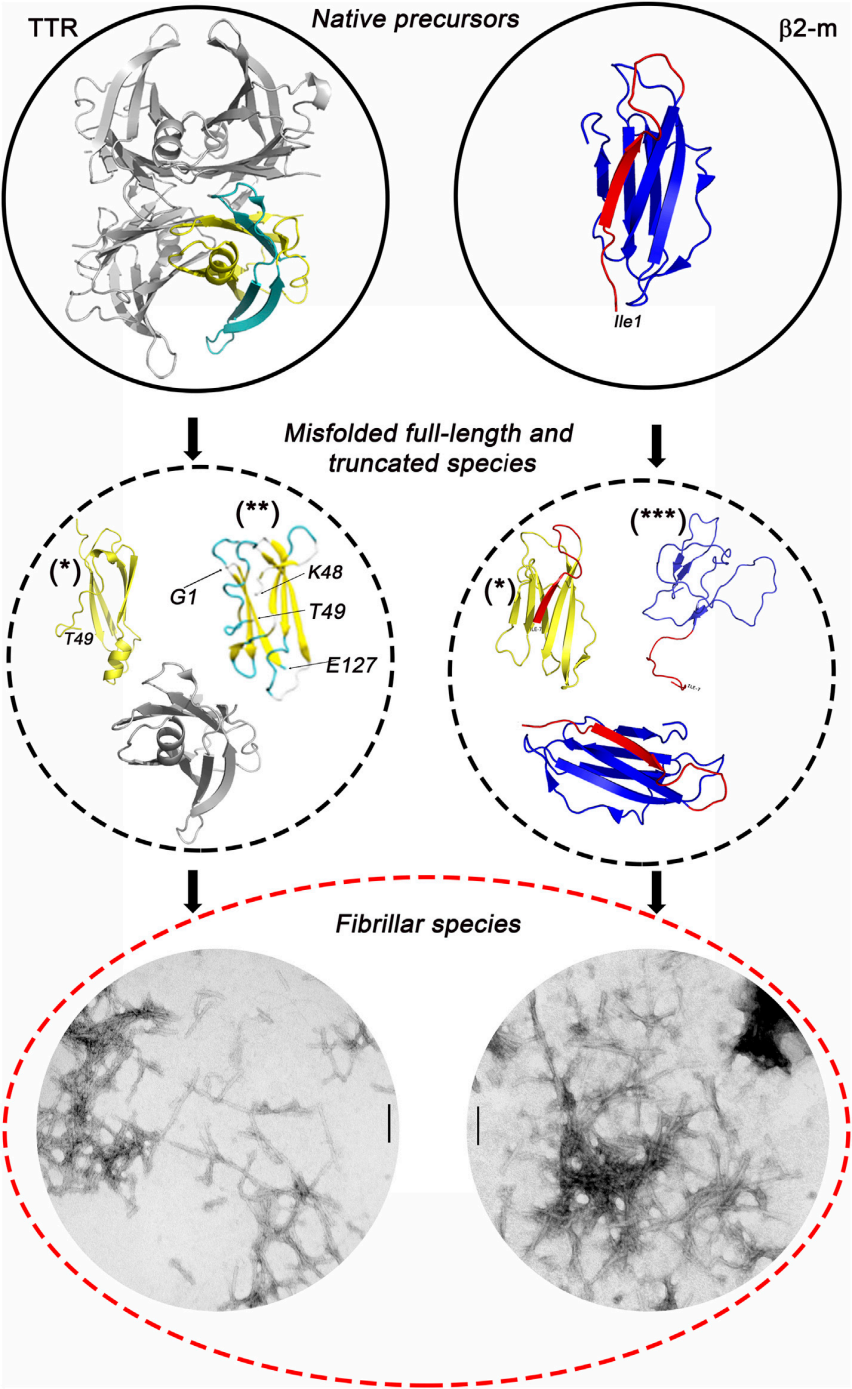
TTR and β2m amyloid formation pathway. Both native proteins may undergo selective proteolytic cleavage generating misfolded amyloidogenic intermediates whose structures still remain elusive even applying advanced structure prediction software such as AlphaFold2. However, despite their different native structure, when misfolded, both TTR and β2-m convert into fibrillar structures that are morphologically similar as shown by negatively stained transmission electron microscopy (scale bar = 100 nm). (*) = structures predicted using the AlphaFold2 software; (**) = conformations predicted using molecular dynamics simulation (Marcoux et al., 2015); (***) = structures derived from NMR spectroscopy measurements (Esposito et al., 2000).

β2-m and TTR (Figure 9) exemplify the need for new paradigms. Along their folding pathway, both these proteins may generate globular or fibrillar structures. Despite their substantial differences in both their primary structure and native state conformation, they reach a similar fibrillar structure at the end of their pathologic transformation. We do not know when the different conformers start moving towards a common β-sheet pathological organization. Elucidation of structure and dynamics of the intermediate states populating the globular-fibrillar transition is of great value. Work on highly amyloidogenic truncated isoforms of β2-m and TTR is very promising and potentially supported by new systems of 3D prediction. However, the attempt to model the conformation of amyloidogenic fragments using AlphaFold2 leads to structures that differ from those based on molecular dynamics or experimental NMR restraints. In the predicted models, a higher proportion of the full-length parent protein is retained possibly because of the use of co-evolutionary methods in AlphaFold2 that may represent a major limitation in predicting the structure of protein aggregation intermediates (Pinheiro et al., 2021). Many proteins can be transformed into amyloid fibrils *in vitro*, but only a few make deposits *in vivo* causing amyloid disease (Chiti and Dobson, 2017). We must recognize that there is still a large gap between what we know about protein amyloidogenesis in experimental models and what happens in patients. Clinical, pathologic studies mirrored by the study of experimental models, which here we have summarized for β2-m and TTR-related amyloidosis, suggest that the disease is multifactorial and occurs only when the amyloidogenic protein acquires a conformation prone to form fibrils under conditions yet to clarify. However, the development of the final quaternary structure of amyloid fibrils is affected, apparently, not only by the protein primary structure but also by the tissue microenvironment that may influence which pathway the protein takes during its fibrillar conversion. Indeed, the recent work using cryo-EM has highlighted differences in the types of filament assembly on TTR fibrils isolated from the heart and vitreous body of the eye in carriers of the same amyloidogenic V30M TTR (Schmidt et al., 2019; Iakovleva et al., 2021).

A better understanding of the effects of the environmental determinants on the conformational dynamics of the protein may provide clues on the structural basis of the intrinsic amyloidogenic property of every specific protein. The complexity of this pathologic process arises through the effect of two dynamic entities. On one hand, we have proteins that can acquire different conformations and, on the other, we have a biological environment under dynamic transformation. In the field of amyloid disease, we are dealing with the very challenging interplay of two entities: protein metamorphosis linked to the organism’s physiology which is influenced by pathological features of environment, disease and age. An amyloidogenic protein and its environment are both in dynamic transformation and we cannot imagine to understand better the one without elucidate the role of the other in the time frame in which we develop the disease.
